# Discriminative Scale Learning (DiScrn): Applications to Prostate Cancer Detection from MRI and Needle Biopsies

**DOI:** 10.1038/s41598-017-12569-z

**Published:** 2017-09-28

**Authors:** Haibo Wang, Satish Viswanath, Anant Madabhushi

**Affiliations:** 1 Philips Research North America, Cambridge, MA 02141 USA; 2 BME Department, Case Western Reserve University, Cleveland, OH 44106 USA

## Abstract

There has been recent substantial interest in extracting sub-visual features from medical images for improved disease characterization compared to what might be achievable via visual inspection alone. Features such as Haralick and Gabor can provide a multi-scale representation of the original image by extracting measurements across differently sized neighborhoods. While these multi-scale features are effective, on large-scale digital pathological images, the process of extracting these features is computationally expensive. Moreover for different problems, different scales and neighborhood sizes may be more or less important and thus a large number of features extracted might end up being redundant. In this paper, we present a Discriminative Scale learning (DiScrn) approach that attempts to automatically identify the distinctive scales at which features are able to best separate cancerous from non-cancerous regions on both radiologic and digital pathology tissue images. To evaluate the efficacy of our approach, our approach was employed to detect presence and extent of prostate cancer on a total of 60 MRI and digitized histopathology images. Compared to a multi-scale feature analysis approach invoking features across all scales, DiScrn achieved 66% computational efficiency while also achieving comparable or even better classifier performance.

## Introduction

A major challenge to overcome in development and application of radiomic and computer assisted decision support methods is to find a way to balance the contrasting requirements of accuracy and computational complexity, especially in the context of very large images such as digital pathology slides^1–7^. Hand-crafted features, such as Gabor Wavelet^8^, Haralick^9^, Scale Invariant Feature Transform (SIFT)^10^, Speeded-Up Robust Features (SURF)^11^, Local Binary Pattern (LBP)^12^ have been shown to be extremely useful for object detection. For example, the LBP descriptor of a pixel is a string of binary bits, each of which is obtained by comparing the gray value of the pixel with pixels within a ring centered on the pixel under consideration.

Computer aided disease detection often needs an exhaustive search of an entire image to accurately match all pixels to the individual disease classes. Salient image descriptions is often a critical pre-requisite for identifying image regions corresponding to disease presence. Finding an appropriate local window size is critical for extracting the salient image descriptions at a pixel-by-pixel level. Too large a window size can substantially increase the computational complexity, while too small a window size could lead to inability in capturing relevant architectural image detail. The conventional solution to the problem is to extract features at a variety of scales, e.g., by performing the same operations at multiple resolutions in a pyramid. When classification is performed, feature matching then takes place at each of the individual scales and the similarity is calculated across the different scales^12^. In this approach, salient feature patterns are under-emphasized due to the sampling of patterns that are irrelevant to disease appearance. Instead of extracting features at many different scales and then matching all of them, it appears more appropriate to only extract features at the most discriminating scales^10,13^. However, the same features or filters extracted at different scales can capture different types of attributes associated with the region of interest (e.g. local edge orientations for a Gabor filter at lower scales and dominant gradient orientations at higher scales). Consequently, the ability to invoke and combine feature responses across different scales will allow for improved discriminability. Conventional approaches that address the problem of discriminative scale selection tend to rely heavily on scale sampling such as dense sampling^12^ or *ad hoc* sampling^14^. There is not, to the best of our knowledge, principled ways to efficiently handle this problem.

In this paper, we present a new Discriminative Scale learning (DiScrn) based approach to tackle the problem of selecting discriminating scales for multi-scale feature extraction from medical images. Unlike existing solutions^12,14^, DiScrn provides a principled way to guarantee that the selected feature scales are the most discriminating. The key idea is to learn a scale weight vector by minimizing the square of similarity distances between positive class samples and jointly maximizing the dissimilarity metrics between positive (cancer) and negative class samples. This results in an optimization problem. Together with the additional constraints that each element of the vector must be in the range of [0, 1] and their sum should sum to one, we obtain a typical convex optimization problem. An iterative solution is presented to resolve this convex optimization problem. In practical real world applications one is only concerned with the testing stage, i.e. how the system and classifier perform in real time. The optimization is no longer needed during the testing stage, since the scales have already been learned.

We evaluate the application of DiScrn in the context of two different prostate cancer detection problems. In the first application, we attempt to use DiScrn for pixel based detection of prostate cancer on multi-parametric MRI. We specifically look at patients who are receiving a staging MRI and subsequently going on to get a radical prostatectomy. Consequently by deformably registering the *in vivo* imaging with the *ex vivo* pathology we are able to spatially map disease extent onto the *in vivo* imaging. This “ground truth” mapping for disease extent allows for training and evaluating the discriminative scale based learning approach for cancer diagnosis. To robustly evaluate our approach we used data from two different institutions, using data from one site to train and data from the other site to validate the DiScrn approach. This is, to the best of our knowledge, the first instance of an attempt to use data from different sites to train and validate a computer aided diagnosis classifier for prostate cancer from multi-parametric MRI. For our second use case, we evaluated DiScrn in terms of identifying regions of cancer on digitized histological slide images of prostate cancer biopsy samples. Pathologist annotations of cancer extent on the digitized biopsy samples was used to train and evaluate the DiScrn approach.

The rest of the paper is organized as follows. In Section II we briefly review previous related work on scale selection and discuss the novelty of our approach. In Section III we describe the discriminative scale learning approach in detail and also explain how the approach was used to construct the classifier. In Section IV we present the experimental results and accompanying discussion for constructing classifiers from MRI and digital pathology images for prostate cancer detection. Section V closes the paper with concluding remarks.

## Related Work and Brief Overview of DiScrn

Scale selection has been a key research issue in the computer vision community since the 1990s^15^. Early investigations in scale selection were based on identifying scale-invariant locations of interest^10,13,16,17^.

Although the idea of locating high interest points is interesting, it is not very feasible for applications where there is a need to investigate every image pixels, e.g., scenarios where one is attempting to identify the spatial location of cancer presence on a radiographic image. In these settings the ability to identify a single, most discriminating scale associated with each individual image pixel is computationally untenable. To address this challenge, Wang *et al*.^18^ presented a scale learning approach for finding the most discriminative scales for Local Binary Patterns (LBP) for prostate cancer detection on T2W MRI.

While a number of recent papers have focused on computer assisted and radiomic analysis of prostate cancer from MRI^19,20^, these approaches typically involve extraction of a number of different texture features (Haralick co-occurrence, Gabor filter, and LBP texture features) to define “signatures” for the cancer and non-cancerous classes. Similarly, some researchers have taken a computer based feature analysis approach to detecting and grading prostate cancer from digitized prostate pathology images using shape, morphologic, and texture based features^2,6,21–23^. However with all these approaches, features are typically either extracted at a single scale or then extracted across multiple scales. Feature selection is then employed for identifying the most optimally discriminating scales^2,3^.

**Figure 1:**
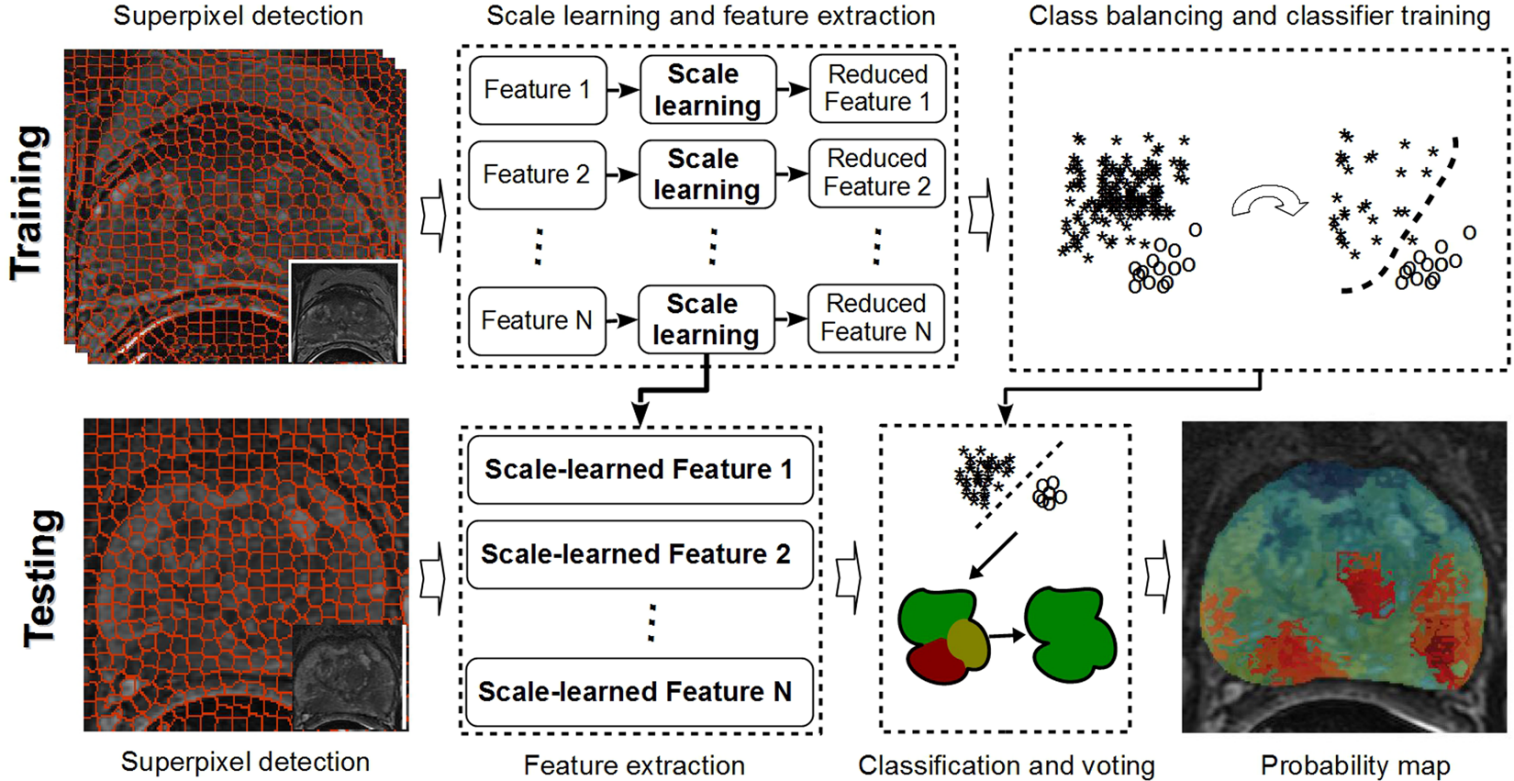
Pipeline of the new DiScrn approach. At the training stage, superpixel detection is performed. An equal number of positive and negative pixels based off the superpixels (see details in Section III.D) are selected during the training phase. Up-to-N different textural features are extracted at various scales for each sampled pixels. For each feature class, its most discriminating scales are learned via DiScrn. Subsequently, a cancer/non-cancer classifier is trained only with the features extracted at the learned scales. At the testing stage, with superpixels detected on a test image, the features and the corresponding scales identified during the learning phase are employed for creating new image representations. Exhaustive labeling over the entire input image is performed to generate a probability map reflecting the probability of cancerous and non-cancerous regions. Majority voting within each superpixel is finally applied to smooth the generated probability map.

In this paper we present a new generalized discriminative scale learning (DiScrn) framework that can be applied across an arbitrary number of feature scales. The conventional dissimilarity measurement for multi-scale feature is to assign a uniform weight to each scale. Based on this weighting idea, DiScrn invokes a scale selection scheme that retain the scales associated with large weights and ignores those scales with relatively trivial weights. Figure 1 illustrates the pipeline of the new DiScrn approach. It consists of two stages: training and testing. At each stage, we first perform superpixel detection on each image to cluster homogeneous neighboring pixels. This greatly reduces the overall computational cost of the approach. At the training stage, we sample an equal number of positive and negative pixels from each of the labeled training images via the superpixel based approach. We subsequently extract four types of multi-scale features for each pixel: local binary patterns (LBP)^12^, Gabor wavelet (Gabor)^8^, Haralick^9^ and Pyramid Histogram of Visual Words (PHOW)^24^. The discriminability of these features has been previously and substantively demonstrated for medical images^2,3^. For each feature type, the corresponding most discriminating scales are independently learned via the DiScrn algorithm.

DiScrn is different compared to traditional feature selection approaches^25–28^ in that DiScrn specifically aims at selecting most discriminative feature scales while traditional feature selection approach aims to directly select the most discriminating subset of features. Both could potentially reduce the number of features, and therefore may significantly reduce the computational burden associated with feature extraction. However, only DiScrn guarantees that only the most predictive feature scales will be used for subsequent feature extraction during the testing phase. This is particularly beneficial for feature extraction in parallel.

Once the DiScrn approach has been applied, texture features will only be extracted at the learned scales for both the classifier training and subsequent detection. In particular, cancerous regions are detected via exhaustive classification over the entire input image. This results in a statistical probability heatmap, where coordinates having higher probabilities represent cancerous regions. Majority voting within each superpixel is finally applied to smooth the generated probability map. To evaluate the performance of DiScrn, multi-site datasets (MRI and histopathology) and testing are employed.

## Detailed Description of Approach

### Superpixel Segmentation

The superpixel algorithm^29^ enables the decomposition of an image into visually homogeneous regions. Among the existing superpixel approaches, the Simple Linear Iterative Clustering (SLIC) is simple and efficient^29^. SLIC is based on a spatially localized version of k-means clustering, in which each pixel is associated with a feature vector and then k-means clustering is performed.

SLIC takes two parameters: the nominal size of superpixels *rSize* and the strength of the spatial regularization parameter *reqStr*. SLIC first divides an image into a grid with step *rSize*. The center of each grid tile is then used to initialize the corresponding k-means algorithm. Finally, the k-means centers and clusters are merged, yielding superpixels. In practice rSize and reqStr must be chosen or found via experimentation.

### Multi-Scale Feature Extraction

Here we describe the four multi-scale hand-crafted features that we utilize in conjunction with DiScrn.

One class of features considered in this work are LBPs^12^. For an arbitrary pixel, the corresponding LBP number is obtained by counting the number of times the intensity of the pixel under consideration is greater or smaller than the intensities of pixels equally spaced within a circle of pre-defined size centered on it. LBP is thus inherently invariant to local gray-scale shift, and can be rotationally-invariant as well. In practice, multi-scale LBP is often used, which consists of a set of single LBP values captured at a family of differently sized circles centered on the pixel of interest. The metric for measuring similarity between a pair of multiscale LBPs is the Hamming distance.

The second family of filters that we consider in this work is the Gabor filter bank^8^. The Gabor filter bank when convolved with an image results in a series of multi-scale, multi-oriented gradient responses. Gabor wavelets are based on Gaussian filters, such that as the distance from the center pixel increases, the value of the function becomes exponentially suppressed. The most critical parameter is the standard deviation *σ* of the Gaussian function, also called the scale factor, which determines the effective size of the neighborhood of the pixel within which the filter response is being measured. A commonly used similarity metric for the Gabor feature is the Euclidean distance.

The third class of features considered in this work is the Haralick feature^9^. This feature is based off a squared gray-level co-occurrence matrix centered at each image pixel. The Haralick feature consists of 14 statistics, capturing different measurements pertaining to the joint intensity distributions within local neighborhoods. The size of the gray-level co-occurrence matrix determines how many adjacent pixels are involved when calculating the statistics. Varying the size of this co-occurrence matrix leads to multi-scale Haralick features. The similarity metric for Haralick features is the Euclidean distance.

The final class of features considered in this work is dense Scale Invariant Feature Transform (SIFT), also known as PHOW (Pyramid Histogram Of visual Words)^24^. SIFT is invariant to geometrical transform, illumination changes and small image distortion. However, the SIFT feature is only suitable for sparse keypoint representation, as it relies on a time-consuming keypoint detection step to determine the optimal image scale. Motivated by this, PHOW was invented to apply SIFT to generate a dense pixel representation in a manner that is computationally efficient. The similarity metric for Haralick feature is also the Euclidean distance.

### DiScrn

Assuming the feature **x** is extracted at *S* different scales, scale selection seeks a vector **w** ∈ *R* ^*S*×1^ such that the dissimilarity metric turns to a weighted sum

1 $$ {\mathcal H} ({\bf{x}},{\bf{x}}^{\prime} )=\sum _{s=1}^{S}{w}_{s}h({{\bf{x}}}_{s},{{\bf{x}}}_{s}^{^{\prime} })={{\bf{w}}}^{T}{\bf{h}},$$

where **x** and **x′** are a pair of features extracted at the *s* th scale. *h*(·) is a basic distance metric defined at each scale, and **h** is a column vector of $h({{\bf{x}}}_{s},{{\bf{x}}}_{s}^{^{\prime} })$. *w*_*s*_ ∈ **w** is the weight of the *s* th feature scale.

Our goal is to learn an optimal **w** from a set ${\mathscr{P}}$ of positive representative samples (extracted from cancer pixels in our case) and a set ${\mathscr{N}}$ of negative multi-scale feature descriptors (non-cancer pixels in our case), by simultaneously minimizing the weighted distance metric between all samples of ${\mathscr{P}}$ and maximizing that distances of samples within ${\mathscr{P}}$ and ${\mathscr{N}}$. Compared to Linear Discriminant analysis, the objective here is not to minimize the distances of all samples in ${\mathscr{N}}$. Hence we get the following objective function to maximize:

2 $$\begin{array}{ll} & \mathop{{\rm{\max }}}\limits_{{\bf{w}}}\frac{{\sum }_{{\bf{x}}\in {\mathscr{P}},{\bf{x}}^{\prime} \in {\mathscr{P}}}{{\bf{w}}}^{T}{\bf{h}}{{\bf{h}}}^{T}{\bf{w}}}{{\sum }_{{\bf{x}}\in {\mathscr{P}},{\bf{x}}^{\prime} \in {\mathscr{P}}}{{\bf{w}}}^{T}{\bf{h}}{{\bf{h}}}^{T}{\bf{w}}},\\ s{\rm{.}}t{\rm{.}} & 1\ge {w}_{s}\ge 0,s=\mathrm{\{1,}\ldots ,S\},{\rm{and}}\,\sum _{s=1}^{S}{w}_{s}=1,\end{array}$$

where 1 ≥ *w*_*s*_ ≥ 0 and ${\sum }_{s=1}^{S}{w}_{s}=1$ enforce that *w*_*s*_ ∈ **w** is a weight. Since there are a large number of pixel samples, we measure the metric distances on a per slice basis. Consequently the objective function becomes

$$\frac{\sum _{t=1}^{Ts}\,\sum _{{{\bf{x}}}_{t}\in {\mathscr{P}},{{\bf{x}}}_{t}^{^{\prime} }\in {\mathscr{P}}}\,{{\bf{w}}}^{T}{{\bf{h}}}_{t}{{\bf{h}}}_{t}^{T}{\bf{w}}}{\sum _{t=1}^{Ts}\,\sum _{{{\bf{x}}}_{t}\in {\mathscr{P}},{{\bf{x}}}_{t}^{^{\prime} }\in {\mathscr{P}}}\,{{\bf{w}}}^{T}{{\bf{h}}}_{t}{{\bf{h}}}_{t}^{T}{\bf{w}}}=\frac{{{\bf{w}}}^{T}{{\bf{H}}}_{b}{{\bf{H}}}_{b}^{T}{\bf{w}}}{{{\bf{w}}}^{T}{{\bf{H}}}_{p}{{\bf{H}}}_{p}^{T}{\bf{w}}},$$

where **H** is now a row matrix of **h** and *T*_*s*_ represents the number of slices. For simplicity, we denote by ${{\bf{S}}}_{p}={{\bf{H}}}_{p}{{\bf{H}}}_{p}^{T}$, and ${{\bf{S}}}_{b}={{\bf{H}}}_{b}{{\bf{H}}}_{b}^{T}$, the intra-class and inter-class distance kernels, respectively. Equation 2 can be arranged in a compact matrix form as,

$$\mathop{{\rm{\max }}}\limits_{{\bf{w}}}\frac{{{\bf{w}}}^{T}{{\bf{S}}}_{b}{\bf{w}}}{{{\bf{w}}}^{T}{{\bf{S}}}_{p}{\bf{w}}},\quad s{\rm{.}}t{\rm{.}}\,{{\bf{b}}}^{T}{\bf{w}}=1,\,{\rm{and}}\,{\bf{0}}\le {\bf{w}}\le {\bf{1}},$$

where ${\bf{b}}={[1,\ldots ,1]}^{T},{\bf{b}}\in { {\mathcal R} }^{S\times 1}$. This is a standard discriminant component analysis problem but with constraints. To solve the problem properly, we follow^30^ to convert the formulation to a least-squares framework:

$$\mathop{{\rm{\min }}}\limits_{{\bf{a}},{\bf{w}}}{\Vert {{\bf{H}}}_{b}^{T}{{\bf{R}}}_{p}^{-1}-{{\bf{H}}}_{b}^{T}{\bf{w}}{{\bf{a}}}^{T}\Vert }_{2}^{2},\quad s{\rm{.}}t{\rm{.}}\,{{\bf{b}}}^{T}{\bf{w}}=1,\,{\rm{and}}\,{\bf{0}}\le {\bf{w}}\le {\bf{1}},$$

where ${{\bf{R}}}_{p}^{T}{{\bf{R}}}_{p}$ is the Cholesky decomposition of **S**_*p*_ and **a** represents the unknown regression coefficients. More details of the intermediate steps and equations can be found in^30^. In practice, the constraint **b** ^*T*^ **w** = 1 may be too restrictive, giving rise to a coarse estimation of **w**. Thus we relax the constraint and instead employ a ${\ell }_{1}-norm$ regularization on **w**, which in turn forces **w** to have a small number of nonzero elements:

3 $$\begin{array}{l}\mathop{{\rm{\min }}}\limits_{{\bf{a}},{\bf{w}}}\,{\Vert {{\bf{H}}}_{b}^{T}{{\bf{R}}}_{p}^{-1}-{{\bf{H}}}_{b}^{T}{\bf{w}}{{\bf{a}}}^{T}\Vert }_{2}^{2}+\eta {\Vert {\bf{w}}\Vert }_{1},\\ \,\,s{\rm{.}}t{\rm{.}}\,{\bf{w}}\ge {\bf{0}},\,{\Vert {\bf{w}}\Vert }_{2}^{2}=1,\,{\Vert {\bf{a}}\Vert }_{2}^{2}=1,\end{array}$$

where the alternative constraint ${\Vert {\bf{w}}\Vert }_{2}^{2}=1$ is to avoid trivial solutions and *η* is a sparsity controller.

Now a new problem is introduced with an extra unknown **a**. Thus problem 3 can be numerically solved by alternating optimization over **a** and **w**.

1. **Solving w given a**: For fixed **a**, **w** is solved by minimizing a LASSO problem:
  4 $$\mathop{{\rm{\min }}}\limits_{{\bf{w}}\ge {\bf{0}}}{\Vert {{\bf{H}}}_{b}^{T}{{\bf{R}}}_{p}^{-1}{\bf{a}}-{{\bf{H}}}_{b}^{T}{\bf{w}}\Vert }_{2}^{2}+\alpha {\Vert {\bf{w}}\Vert }_{2}^{2}+\eta {\Vert {\bf{w}}\Vert }_{1}.$$
  This problem is easily transformed to a sparse nonnegative least-squares (SNNLS) problem
  5 $$\mathop{{\rm{\min }}}\limits_{{\bf{w}}\ge {\bf{0}}}{\Vert (\begin{array}{c}{{\bf{H}}}_{b}^{T}\\ \sqrt{\alpha }{{\bf{I}}}_{S}\end{array}){\bf{w}}-(\begin{array}{c}{{\bf{H}}}_{b}^{T}{{\bf{R}}}_{p}^{-1}{\bf{a}}\\ {{\bf{0}}}_{{N}_{b}\times 1}\end{array})\Vert }_{2}^{2}+\eta {\Vert {\bf{w}}\Vert }_{1}.$$
  The SNNLS problem can be efficiently solved using an existing solver such as the block principal pivoting algorithm^31^.
2. **Solving a given w**: For fixed **w**, the optimal **a** is obtained as

6 $${\bf{a}}=\frac{{{\bf{R}}}_{p}^{-T}{{\bf{S}}}_{b}{\bf{w}}}{\sqrt{{{\bf{w}}}^{T}{{\bf{S}}}_{b}{{\bf{S}}}_{b}{\bf{w}}}}.$$

Given the learned scale weights **w**, we define a threshold *σ*, 0 < *σ* < 1, to automatically determine the number of selected scales. Only the top scales whose weight scores are no smaller than *σ* × *w*_*max*_ will be selected, where *w*_*max*_ is the largest weight value. The threshold, *σ*, can be determined empirically on a test set.

### Imbalanced Classifier Training

**Figure 2:**
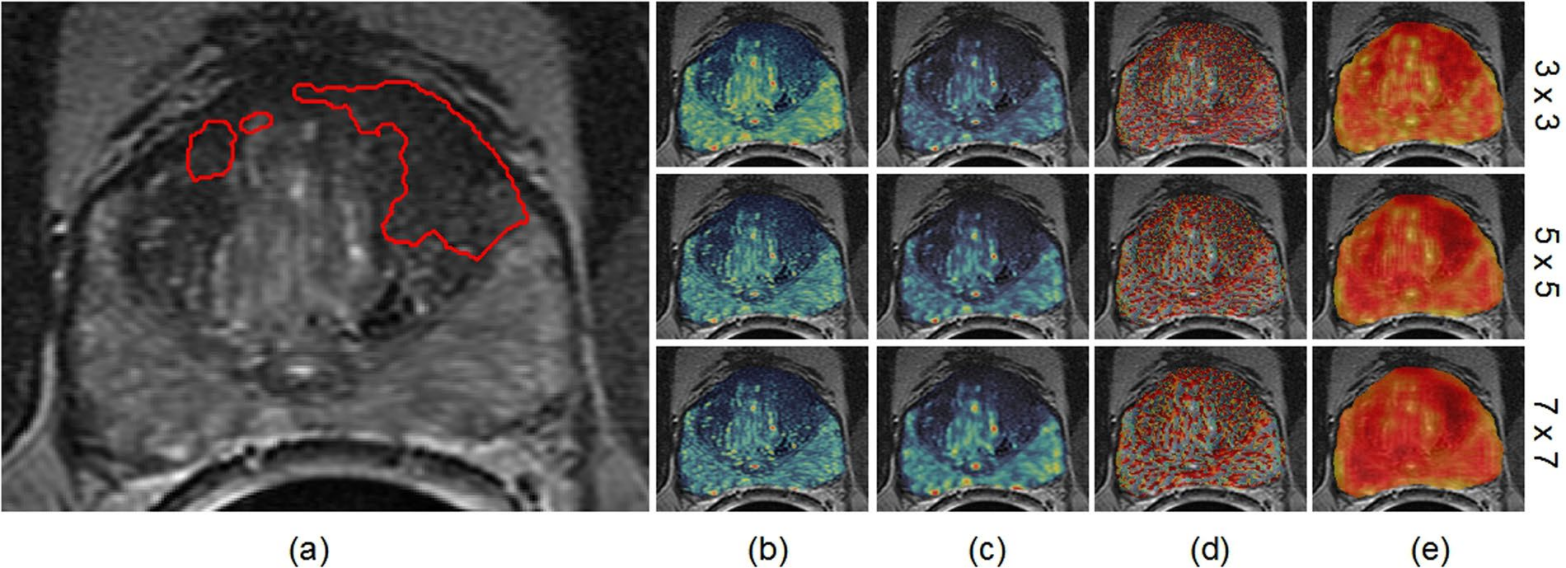
Visualization of multi-scale (**b**) Gabor, (**c**) Haralick, (**d**) LBP and (**e**) PHOW features. Each feature is extracted at the same three scales: 3 × 3, 5 × 5, and 7 × 7. As compared to the (**a**) cancer ground truth, we can see that (1) no scale pattern is obviously more discriminative than the others, and (2) no individual feature appears to perfectly distinguish cancer and benign regions. Therefore, it is necessary to combine all these features and learn the most discriminating scales for each of the features.

A major issue from a classification perspective is the relatively small number of cancer pixels compared to non-cancer pixels (see an example in Fig. 2). This causes a serious classification bias when training a classifier with an imbalanced learning set. There are two typical ways to address this issue in the machine learning literature^32^. One is to assign distinct costs to training examples while the other is to re-sample the original dataset, either by over-sampling the minority class and/or under-sampling the majority class^33^. While randomly under-sampling the majority class is the simplest and most popular approach, it cannot guarantee that the sampled instances are actually independent. Hence repeated under-sampling is often required.

Here we replace the random re-sampling with a smarter strategy based off superpixels. Since superpixels represent a cluster of homogeneously appearing pixels, we sample only one pixel from each superpixel of the majority class to minimize the dependency on the sampled data. In order to train a balanced classifier, we (1) include all the cancer pixels in the positive training class, (2) utilize all the centers of superpixels of non-cancer regions representing the negative training class, and (3) randomly sample some non-cancer pixels to balance the number of positive and negative instances.

With a balanced training set, a Random Forest classifier^34^ is finally trained for distinguishing cancer pixels from the non-cancerous ones.

### Heatmap Smoothing via Superpixels

Multiple features at the learned scales are extracted for each pixel in a testing image. The pixel is then classified as cancer or non-cancer with a probability generated by the trained RF classifier. Generally it is safe to assume that spatially-adjacent and texturally-similar pixels should have similar probabilities. Therefore, we take advantage of the superpixel algorithm to smooth the generated probability map.

**Figure 3:**
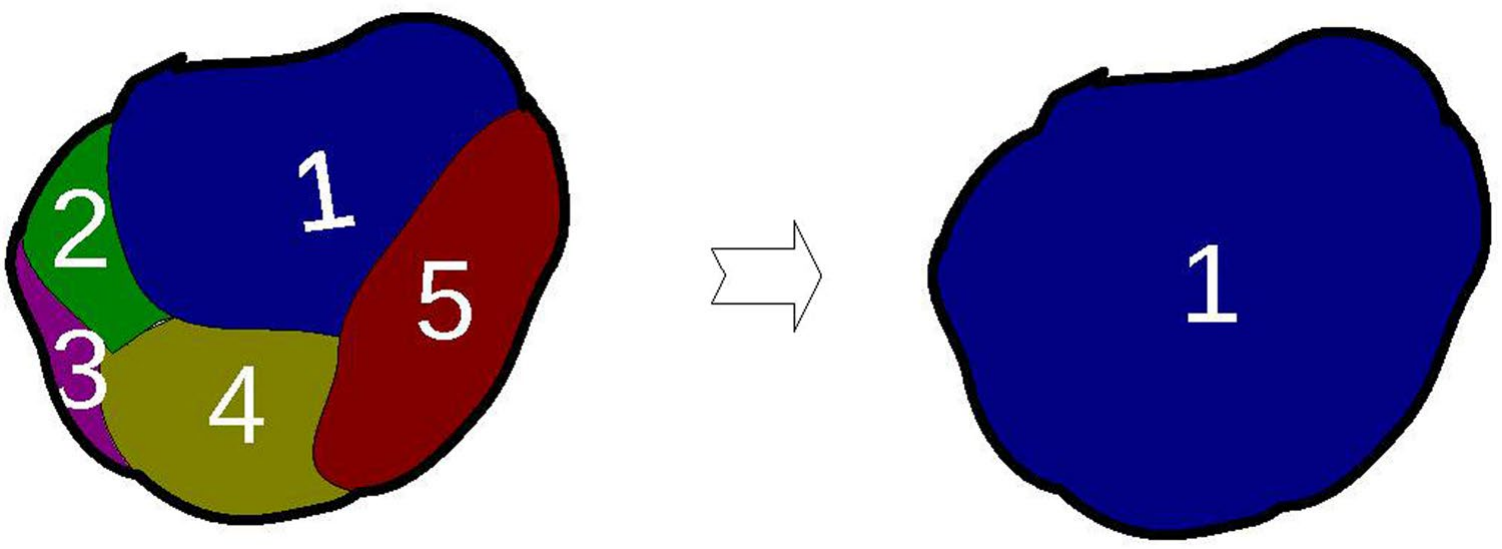
Superpixel-wise voting. As shown, the class probability of all pixels within each superpixel is unified based on majority voting.

Let *V* denote an arbitrary superpixel, consisting of *n* pixels *p*_*i*_ ∈ *V*. Suppose each pixel *p*_*i*_ has a probability value *c*_*i*_ ∈ {0, 0.1, 0.2, …, 0.9, 1}, with 0 representing non-cancer and 1 cancer, the class of the superpixel *V* is determined as

7 $${c}_{v}={\rm{\arg }}\mathop{{\rm{\max }}}\limits_{c}\sum _{i}^{n}sign({c}_{i}=c),\,c\in \{0,0.1,0.2,\ldots ,0.9,1\},$$

which is the probability value associated with a majority of the pixels. In this way, all pixels within a superpixel are forced to have a single probability value, yielding a smoother probability map. Figure 3 illustrates this super-pixel voting idea.

## Experimental Results

### Data Description

DiScrn was evaluated on two different use cases. Details of the datasets are summarized in Table 1 and described below.

**Table 1:** Summary of the datasets employed in this study.

Dataset	Site	Modality	#Patients	Resolution
D1	S1	T2w MRI	16	512 × 512
D2	S1	ADC MRI	16	320 × 320
D3	S2	T2w MRI	22	256 × 256
D4	S2	Histology	22	1100 × 1000

The T2w and ADC MRI in D1 and D2 come from the same S1 site. The T2w MRI in D3 comes from the different S2 site. The MRI imaging of D1, D2 and D3 was performed with 3 mm, 4 mm and 3 mm slice thickness, respectively.

#### Ethics Statement

Data analysis was waived review and consent by the IRB board, as all data was being analyzed retrospectively, after de-identification. All experimental protocols were approved under the IRB protocol #02-13-42C with the University Hospitals of Cleveland Institutional Review Board, and all experiments were carried out in accordance with approved guidelines. Under this IRB, we were allowed to obtain de-identified images from St Vincent’s Hospital and University of Pennsylvania,and material transfer agreements were signed and agreed upon between Case Western Reserve University and University of Pennsylvania and St. Vincent’s Hospital.

#### Prostate MRI

Three different sets of MRI scans were employed in this study. **D1**: This dataset consists of *in vivo* T2w MRI images collected from 16 patients diagnosed with prostate cancer via core needle biopsies. The axial T2w imaging was performed with 3 mm slice thickness. Imaging FOV (field of view) was 14 cm. **D2**: This dataset comprises ADC (Apparent Diffusion Coefficient) MRI prostate images corresponding to the same set of 16 patients listed in D1. Each ADC study comprised roughly 55 slices and with an X-Y plane resolution of 320 × 320 pixels. Imaging FOV was 24 cm. The ADC imaging was performed with 4 mm slice thickness. **D3**: This dataset comprises T2w MRI prostate scans from 22 prostate cancer patients. The axial T2w imaging was performed with 3 mm slice thickness and 1.0 mm gap. Imaging FOV (field of view) was 14 cm, and acquisition matrix size was 256 by 128–179.

The surgically resected prostate gland, after fixation in formalin, was sectioned in a plane perpendicular to the urethral axis from apex to base into 3–4 mm slices. Each slice was then divided into 4 quadrants, stained with H&E and digitized by the Aperio whole slide scanner at 20× magnification. The goal of this study was to distinguish between cancer and benign regions on a per pixel basis on T2w MRI. In this study we focused on patients with prostate cancer who were undergoing radical prostatectomy and had a staging MRI done. The advantage of using surgical patients was that we had access to the *ex vivo* pathology and the *in vivo* imaging which in turn allowed us to co-register and hence map spatial extent of the cancer from the *ex vivo* histopathology onto the *in vivo* imaging. The co-registration of the pathology and *in vivo* imaging was done using the approaches described in^35,36^ and briefly described below.

1. Correspondences between MRI and H&E stained histological slices were jointly identified by an expert radiologist and pathologist working together, employing distances between slices and major anatomical landmarks;
2. Histological sections are first registered to MRI slices by using thin plate splines (TPS)^36^, which maximizes the overlap between the target and template landmarks. This procedure helps establish accurate spatial correspondences while minimizing the bending energy to generate smooth transformations;
3. Manually selected landmarks are used to align the boundaries of prostate on the mapped histological and MR images;
4. Spatial extent of cancer on histology is mapped onto corresponding MRI sections.

#### Prostate Biopsy Core Samples

The goal of this study was to detect cancer regions on a per pixel basis from digitalized images of prostate core needle biopsy specimens. This dataset (**D4**) comprises digitized images of H&E stained histological prostate biopsy images from 22 patients. Tumor area on the digital slide was annotated by expert pathologist.

### Experimental Design

**Figure 4:**
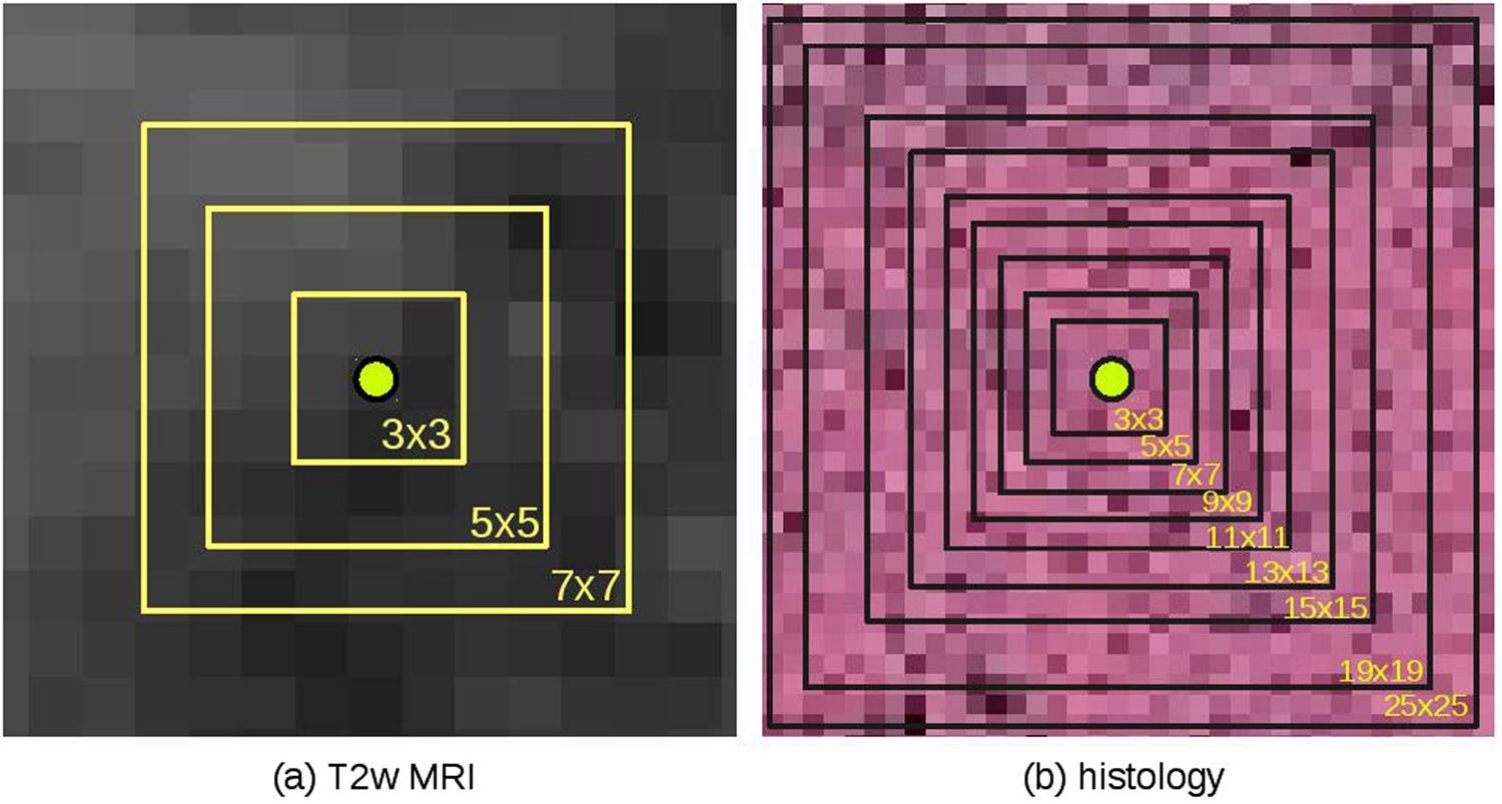
Illustrating the used feature scales for MRI and histology. Three feature scales - 3 × 3, 5 × 5, 7 × 7 - are used for feature extraction on the low-resolution MRI images of D1, D2 and D3. For the high-resolutional histological images, up-to 9 feature scales are used: 3 × 3, 5 × 5, 7 × 7, 9 × 9, 11 × 11, 13 × 13, 15 × 15, 19 × 19, and 25 × 25.

The four feature classes (Gabor, Haralick, LBP and PHOW) used in this work were extracted at multiple scales for distinguishing cancerous from non-cancerous regions. Owing to the size of the MRI scans in D1, D2 and D3, only 3 feature scales (3 × 3, 5 × 5, and 7 × 7) were employed. On the high-resolutional histological images in D4, up to 9 feature scales are used: 3 × 3, 5 × 5, 7 × 7, 9 × 9, 11 × 11, 13 × 13, 15 × 15, 19 × 19, and 25 × 25. Figure 4 illustrates how the feature scales were defined for the MRI and histopathology datasets.

DiScrn was compared with the scheme using all predefined feature scales (termed as **AllScales**) and T-test features selected from all predefined feature scales (termed as **T-test**). Two different evaluation metrics were employed for evaluating DiScrn and comparative approaches. First, the cancer detection accuracy of the two schemes are compared in terms of their resulting AUC (area under ROC (Receiver Operating Characteristic) curve) values. Second is the time required to extract the four feature classes at the testing stage. Each experiment was repeated ten times and the average AUC values and computational times at run-time reported. To systematically evaluate DiScrn, we designed the following various experiments:

- **E1** (D1) - randomized *K*-fold cross validation on D1 with *K* ∈ {2, 4, 6}. The goal of this experiment was to evaluate the scalability of DiScrn on T2w MRI as a function of various training set sizes.
- **E2** (D2) - randomized *K*-fold cross validation on D2 with *K* ∈ {2, 4, 6}. The goal of this experiment was to evaluate the scalability of DiScrn on ADC MRI as a function of various training set sizes.
- **E3** (D1 + D2) - randomized *K*-fold cross validation on the combination of D1 and D2 with *K* ∈ {2, 4, 6}. The goal of this experiment was to evaluate the scalability of DiScrn on T2w + ADC MRI as a function of various training set sizes.
- **E4** (D1 → D3) - using D1 for training and D3 for testing.
- **E5** (D3 → D1) - using D3 for training and D1 for testing.

**E6** - randomized *K*-fold cross validation on D4. *K* was fixed to be 5, i.e., randomly selecting 80% of the 22 studies in D4 for training and using the remaining cases for testing.

All the experiments were conducted within a Matlab environment on a 64-bit Linux machine with 4-core CPU and 4G memory. On all the experiments, different values of the key parameters were tested on a smaller set and thereafter kept constant. *rSize* and *reqStr* are the two critical parameters for superpixel segmentation. For all experiments we empirically set the parameters as *rSize* = 8 and *reqStr* = 0.01. The regularization controllers in Eq. (6) were set as *α* = 0.1 and *μ* = 0.001. The feature selection threshold *σ* is set at 0.3. The angles for the Gabor filter bank were set to 0°, 90° and 180°. The bit length for the LBP feature at each scale is set to 8. The PHOW length at each scale is set to 128. The number of trees employed in the random forest classifier is set to 50. Different values of *σ* were experimented with on a smaller set of cases and the optimal value locked down as 0.3 and employed for all subsequent experiments.

### Results of DiScrn in detecting cancer on Prostate MRI scans

**Figure 5:**
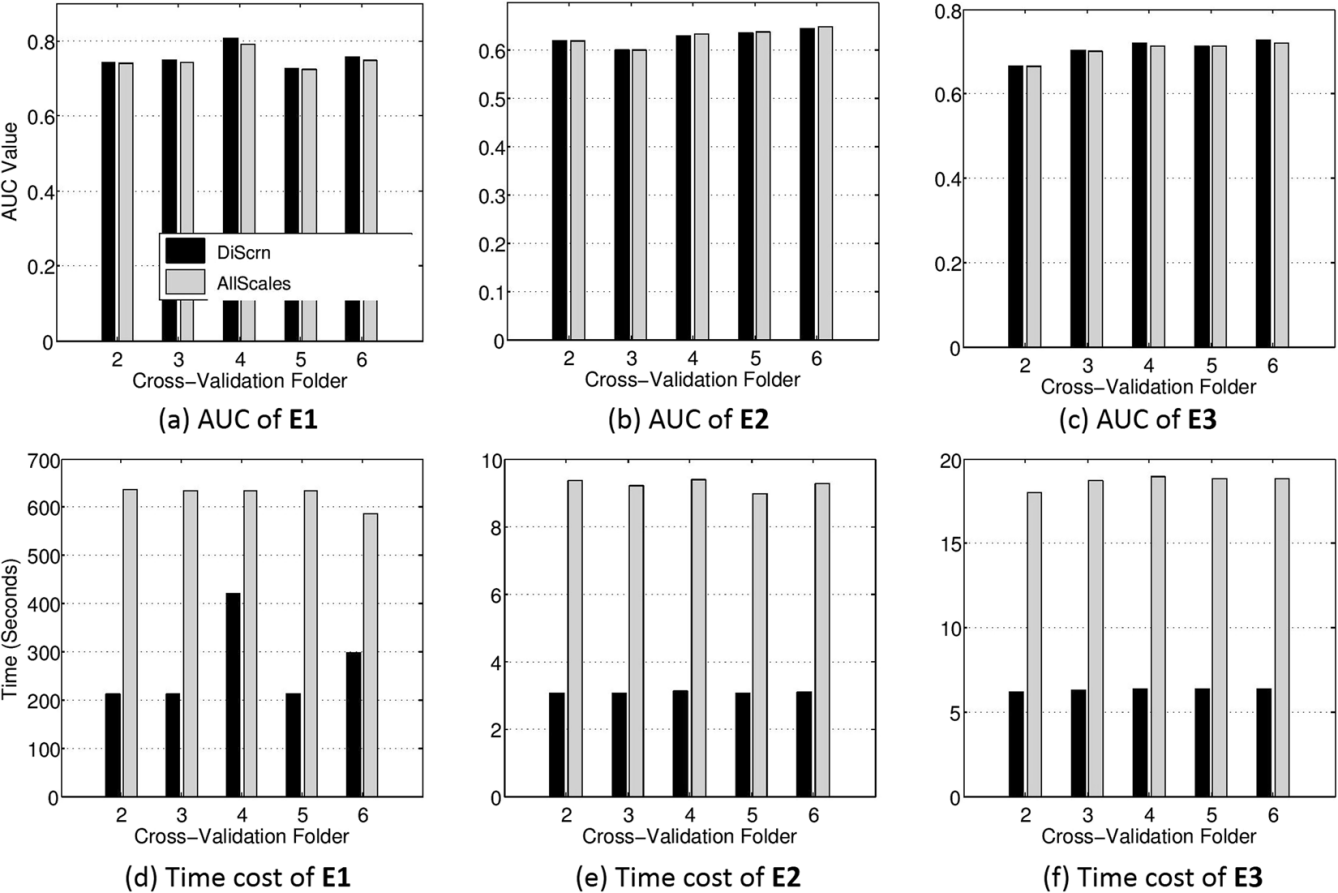
Experimental results of the **E1**, **E2** and **E3** experiments. (**a**–**c**) are the resulting AUC values of the three experiments. (**d**–**f**) are the per-slice feature extraction time at the testing stages of these experiments.

**Figure 6:**
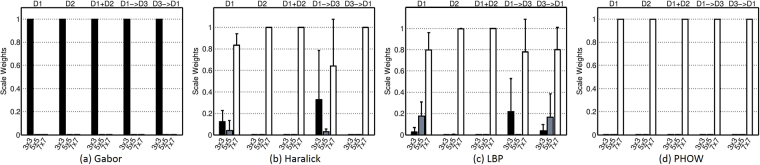
Examplar heatmaps (%) of cancer detection on T2w MRI images for the **E1** experiment. The color bar reflects the corresponding probability value. Higher probability indicates more likely cancer region. Given the (**a**) ground truth, the presented scale learning approach leads to (**c**) slightly more accurate cancer heatmaps as compared to (**b**) the results of using all predefined feature scales. Note that the feature extraction time of DiScrn is only about 1/3 of using all predefined feature scales (AllScales).

We begin by noting that the objective of the experiments in this study was not that DiScrn yields the best possible prostate cancer detection classifier on MRI and histopathology. Instead we seek to show that comparable accuracy can be obtained (along with substantial computational efficiency) in employing features only at a subset of scales. Figure 5 shows the AUC performance for **E1**, **E2** and **E3**, respectively. DiScrn not only significantly reduces the time required for feature extraction at the testing stage, but also slightly improves the accuracy of cancer detection for most cases. While this is somewhat surprising, since intuitively one would expect that employing all the scales should outperform a classifier that only uses features at a subset of scales, DiScrn might be suppressing features at certain scales that negatively contribute to the classification results of the multi-scale (all scale) approach. Note that the lower AUC values for the ADC images in **E2** as compared to that of T2w MRI in **E1** might be on account of the lower resolution (320 × 320) of the ADC MRI. This may also explain why combining T2w and ADC MRI in **E3** is worse than using T2w MRI alone (**E1**), since we physically resize the T2w MRI from 512 × 512 to 320 × 320. However, clearly combining T2w MRI and ADC features (**E3**) improves performance compared to using ADC features alone (**E2**). Figure 6 shows examplar cancer detection heatmaps generated for the **E1** experiment. With DiScrn, the cancer region is more likely to be correctly detected, which is also reflected in the AUC values shown in Fig. 5. Table 2 summarizes the average time cost of feature extraction at the testing stage (*E*3). Clearly the computational cost associated with each feature is greatly reduced, once the most critical discriminating scale has been identified.

**Table 2:** Detailed time cost of feature extraction at the testing stage of the **E3** experiment.

Feature	DiScrn (seconds)	AllScales (seconds)
Gabor	0.12 ± 0.02	0.56 ± 0.05
Haralick	5.64 ± 0.16	16.35 ± 0.55
LBP	0.05 ± 0.01	0.11 ± 0.03
PHOW	0.52 ± 0.05	1.65 ± 0.13

The mean and standard deviations are calculated over all the *K* folds of cross validation.

Table 3 summarizes the results of the cross-site **E4** and **E5** experiments. In particular, we perform a baseline comparison that uses the intensity value of each pixel for the purposes of prediction. Since D1 and D3 come from different sites and have different image sizes, the AUC values of *E*4 and *E*5 are generally lower than the results of **E1**–**E3** (see Fig. 5). However, the scale learning approach still significantly reduces the time cost and improves the overall AUC result. The most significant AUC improvement appears in **E5**.

**Table 3:** Summary of the cross-site **E4** and **E5** experiments.

Method	Feature scales	AUC	Feature extraction time per slice (seconds)
DiScrn	Gabor: 3 × 3, LBP: 5 × 5	**E4**: 0.656	16.60
	Haralick: 5 × 5, PHOW: 7 × 7		
	Gabor: 3 × 3, LBP: 5 × 5	**E5**: 0.657	214.46
	Haralick: 5 × 5, PHOW: 7 × 7		
T-test	Gabor: 3 × 3, 5 × 5, 7 × 7	**E4**: 0.613	N/A
	LBP: 3 × 3, 5 × 5, 7 × 7		
	Haralick: 3 × 3, 5 × 5, 7 × 7	**E5**: 0.618	N/A
	PHOW: 3 × 3, 5 × 5, 7 × 7		
AllScales	Gabor: 3 × 3, 5 × 5, 7 × 7	**E4**: 0.640	28.54
	LBP: 3 × 3, 5 × 5, 7 × 7		
	Haralick: 3 × 3, 5 × 5, 7 × 7	**E5**: 0.603	631.11
	PHOW: 3 × 3, 5 × 5, 7 × 7		
Baseline	Intensity: 1 × 1	**E4**: 0.518	≈0.0
		**E5**: 0.475	

DiScrn is compared with using all predefined feature scales, T-test features selected from all predefined feature scales, and a baseline scheme that uses raw pixel intensities. The evaluation metrics are the AUC and the feature extraction time per slice at the testing stage. The second column shows the feature scales used for feature extraction. Note that we did not report the run time of T-test for feature extraction at the testing stage. This is because we did the feature extractions in parallel across all scales. In practice, feature selection can save the extraction time.

**Figure 7:**
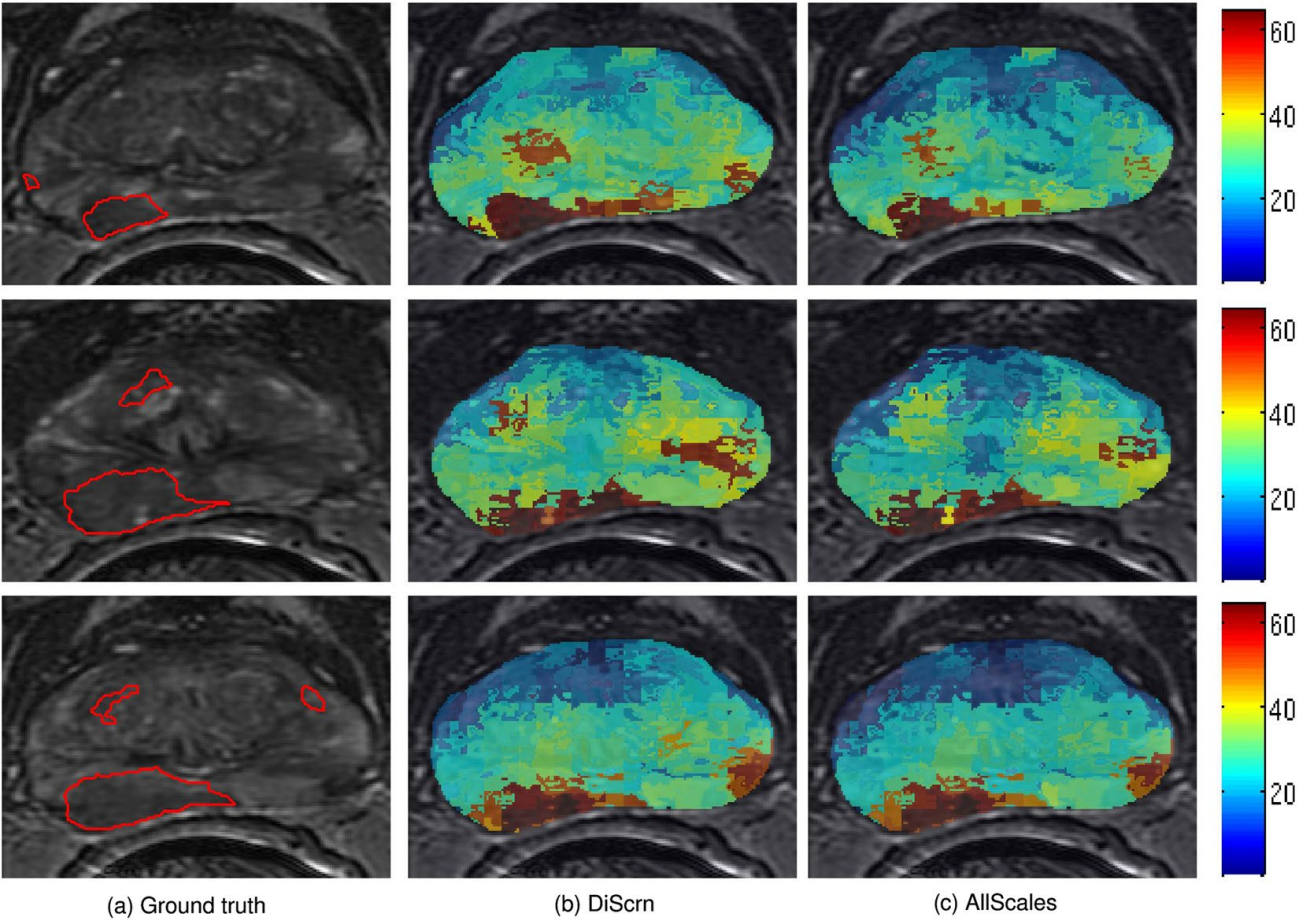
The learned scale weights (mean + std) of (**a**) Gabor, (**b**) Haralick, (**c**) LBP, and (**d**) PHOW on MRI images from the experiments of **E1** (D1), **E2** (D2), **E3** (D1 + D2), **E4** (D1 → D3) and **E5** (D3 → D1). The horizontal axis represents the used three feature scales (i.e., 3 × 3, 5 × 5, 7 × 7) for the five different experiments. The vertical axis represents the learned weights. High weight indicates high importance and vice versa.

Figure 7 summarizes the weight distributions for each of the individual scales learned from experiments **E1**–**E5**. The most discriminating scales identified for Gabor, LBP and PHOW were 3 × 3, 7 × 7, and 7 × 7, respectively. These results appear to suggest that the Gabor filter bank allows for capture of local features while the LBP and PHOW tend to capture larger macro level features that best discriminate between cancerous and non-cancerous regions. In most cases, 7 × 7 is the sole discriminative scale for the Haralick feature, but 3 × 3 is also selected when D1 is used for training and D3 for testing. In our analysis, since the X-Y image plane size (512 × 512) of D1 is double of that of D3 (256 × 256), a scale size of 3 × 3 in D1 approximately corresponds to the 7 × 7 scale size for the studies in D3. Moreover, the consistency of the weights for the individual scales reveals that DiScrn appears to be robust to the size and variability within the training set. Note that while the AUC results for DiScrn for prostate cancer detection on MRI appear to be lower than what has been reported by other groups including our own^37–40^, it needs to be stressed that our results should not be directly compared against these previous findings. The reasons are on account of the fact that (a) these previous studies primarily focused on multi-modal fusion (e.g. DCE MRI, MR Spectroscopy, T2w, Diffusion) and (b) none of these approaches looked at cross-site validation and limited to training and evaluating their classifiers to data from a single site. However in spite of these competititve disadvantages, DiScrn still manages to provide high accuracy for voxel-level classification on individual imaging protocols and more importantly provides consistent and highly efficient classification results across multi-institutional data. We argue that further improvement in accuracy will most likely be driven by identification and choice of new features. This is not the stated goal of this work which is to take existing handcrafted features and improve their efficiency. All things being equal, our major contribution is that without compromising on accuracy we are able to provide substantial improvements in efficiency for the problem of prostate cancer detection on MRI as well as digitized pathology images. This was evidenced by the extensive evaluation on two different use cases and with independent training and testing datasets.

### Results on Histological Images

**Figure 8:**
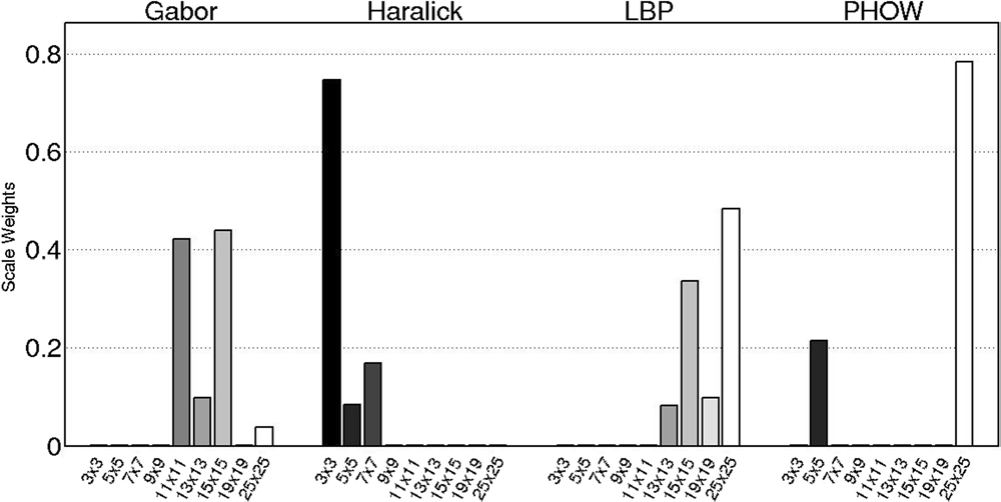
The learned scale weights of (**a**) Gabor, (**b**) Haralick, (**c**) LBP, and (**d**) PHOW on histological images used in **E6**. The horizontal axis represents the evaluated 9 feature scales, i.e., 3 × 3, 5 × 5, 7 × 7, 9 × 9, 11 × 11, 13 × 13, 15 × 15, 19 × 19 and 25 × 25. The vertical axis represents the learned weight distributions of the 9 feature scales. Note that only a subset of scales were identified as being important for this problem.

Figure 8 shows weights learned via the DiScrn approach for the individual scales of the different feature classes in **E6**. Table 4 summarizes the cancer detection results for DiScrn and the multi-scale approach for **E6**. The weight distributions in Fig. 8 appear to suggest that only a small subset of scales are contributory to the final prediction. For *σ* = 0.3, the selected scales for Gabor, Haralick, LBP and PHOW are shown in Table 4. Each feature class appears to have its own discriminative scale patterns, which implies that manually selecting the optimal scale for each feature is difficult. Table 5 shows that as a result of using the scales selected by DiScrn, the feature extraction time is significantly reduced by about 60%. Meanwhile, the AUC value increases from 0.826 to 0.836. Note that these classification results are actually comparable and even superior to results previously reported for this problem^2^.

**Table 4:** Cancer detection results on the histological images from the **E6** experiment.

Method	Feature scales	AUC	Extraction time (seconds)
AllScales	Gabor: 3 × 3, 5 × 5, 7 × 7, 9 × 9, 11 × 11, 13 × 13, 15 × 15, 19 × 19, 25 × 25	0.826	4644.38
	LBP: 3 × 3, 5 × 5, 7 × 7, 9 × 9, 11 × 11, 13 × 13, 15 × 15, 19 × 19, 25 × 25		
	Haralick: 3 × 3, 5 × 5, 7 × 7, 9 × 9, 11 × 11, 13 × 13, 15 × 15, 19 × 19, 25 × 25		
	PHOW: 3 × 3, 5 × 5, 7 × 7, 9 × 9, 11 × 11, 13 × 13, 15 × 15, 19 × 19, 25 × 25		
T-test	Gabor: 3 × 3, 5 × 5, 7 × 7, 9 × 9, 11 × 11, 13 × 13, 15 × 15, 19 × 19, 25 × 25	0.764	N/A
	LBP: 3 × 3, 5 × 5, 7 × 7, 9 × 9, 11 × 11, 13 × 13, 15 × 15, 19 × 19, 25 × 25		
	Haralick: 3 × 3, 5 × 5, 7 × 7, 9 × 9, 11 × 11, 13 × 13, 15 × 15, 19 × 19, 25 × 25		
	PHOW: 3 × 3, 5 × 5, 7 × 7, 9 × 9, 11 × 11, 13 × 13, 15 × 15, 19 × 19, 25 × 25		
DiScrn	Gabor: 11 × 11, 15 × 15	0.836	1343.17
	LBP: 3 × 3, 7 × 7		
	Haralick: 15 × 15, 19 × 19, 25 × 25		
	PHOW: 5 × 5, 25 × 25		

AUC and Computational costs are reported for DiScrn and the all scale based approach while only AUC is reported for using T-test features selected from all predefined feature scales. Also shown are the most predictive scales identified for different features by DiScrn. Note that we did not report the run time of T-test for feature extraction at the testing stage. This is because we did the feature extractions in parallel across all scales. In practice, feature selection will save the extraction time.

**Table 5:** Detailed time cost of each feature extraction approach on the high-resolutional histological images used in **E6**.

Features	AllScales (seconds)	DiScrn (seconds)
Gabor	9.62	2.49
Haralick	4352.98	1308.32
LBP	1.08	0.50
PHOW	280.70	31.86

For each experiment involving statistical significance testing, we first counted the total number of features selected via DiScrn. Then we forced the T-test based selection method to select the same number of features. Since the information of feature scales is not implicitly considered by T-test, all feature scales tend to be selected by T-test. However, as shown in Tables 3 and 4, DiScrn outperforms T-test in terms of AUC value.

### Concluding Remarks

This paper presented a discriminative feature scale learning (DiScrn) approach to address the issue of finding and combining the optimal scales at which the most discriminating features could be identified. We evaluated DiScrn on two different problems relating to prostate cancer detection, one involving MRI from two different sites and the other involving digital pathology images. By learning a vector that weighs the discrimination score at each individual scale, DiScrn allows for computation of a metric that better represents the target class. When evaluated on the two different use cases considered in this work, DiScrn was able to improve the accuracy of cancer prediction as compared to an approach that attempted to combine features from all possible scales. Most critically, DiScrn significantly reduces the computational time associated with feature extraction, especially during the testing phase. Our main findings in using DiScrn were that (1) different feature classes tend to be most discriminating at unique scales (i.e. there is no single magic scale at which all features tend to be most discriminating), and (2) using features at only the most discriminating scales results in classification performance that is comparable and in many cases superior to an approach that employs features from across all scales.

A key strength of this work was that DiScrn was rigorously and robustly evaluated on different problems, different imaging modalities and sequences, different image resolutions, and most critically using data from different institutions. Our results showed that DiScrn was robust to the size and variety of the training sets and achieved consistent prediction results when data from different sites were used for training and independent testing. This work represents to the best of our knowledge the first attempt to evaluate a machine learning approach for prostate cancer detection from multiple different sites^37–40^. Moreover, we observe that (1) in MRI images it is sufficient to select only one scale for each feature type, and (2) in large-scale histological images, each feature was found to be discriminating across several scales.
